# High-Temperature Passivation of the Surface of Candidate Materials for MSR by Adding Oxygen Ions to FLiNaK Salt

**DOI:** 10.3390/ma15155174

**Published:** 2022-07-26

**Authors:** Eduard A. Karfidov, Yuri P. Zaikov, Evgenia V. Nikitina, Konstantin E. Seliverstov, Alexey V. Dub

**Affiliations:** 1 The Institute of High Temperature Electrochemistry of the Ural Branch of the Russian Academy of Sciences, 620066 Yekaterinburg, Russia; karfused@mail.ru (E.A.K.); dir.zaikov@mail.ru (Y.P.Z.); gluk222@yandex.ru (K.E.S.); 2 National University of Science and Technology MISIS, 119049 Moscow, Russia; alvdub@rosatom.ru

**Keywords:** corrosion, candidate materials for MSR, FLiNaK melt, high-temperature passivation, protective oxide layers

## Abstract

The problem of tailoring the structural materials for MSR is solved by continuously overcoming the shortcomings of widely used materials and finding new ones. The materials commonly used in engineering may not be applicable for MSR due to their high corrosivity. Experiments were carried out to determine the corrosion rate of stainless steel 12Cr18Ni10Ti with different concentrations of oxide ions (by adding lithium oxide to the melt in the concentration range from 0 to 0.8 wt.%) in a FLiNaK melt. The formation of a protective oxygen-containing layer with a thickness of 1 micron has been realized. The corrosion rate decreases by an order of magnitude at the concentration of oxygen anions in the melt, in the range from 0.2 to 0.4% by weight, which may indicate high-temperature passivation of the material due to modification of the composition of the fluoride melt and reduction in its corrosion activity. In addition, the corrosion type of stainless steel in fluoride melts changes from the intercrystalline and pitting that is usually harmful to reactor material structure to total corrosion when lithium oxide is added. This is due to the “healing” of individual corrosion defects formed on the surface of the studied material by oxygen-containing compounds.

## 1. Introduction

Molten fluoride salts have properties that allow them to be used as salt solvents in a liquid salt reactor [1,2,3,4,5].

Despite the fact that alkali metal fluorides are among the most aggressive salts in terms of degradation of construction materials used in reactor engineering, these salt melts have huge advantages over water solutions due to high thermal conductivity, low viscosity, high boiling points, the highest heat capacity per unit volume and insensitivity to radiation.

The great advantages of the designs of the IV generation reactor systems with molten salts are the efficient use of fuel, minimal amount of radioactive waste and expected economically attractive, safe and environmentally friendly operation [6,7,8]. Molten salts can be used as a reactor coolant or transfer medium in high-temperature technological thermal circuits (from nuclear reactors to hydrogen production), but corrosion of metallic materials is a serious problem [9,10,11].

Attempts to reduce the rate of corrosion in molten salt systems face serious, sometimes insurmountable limitations. In many high-temperature installations where molten salts are applied, alloys containing high concentrations of chromium, silicon or aluminum are traditionally used, since these elements easily form passive oxide films that slow down the transfer of particles between the metal and the environment, preventing further corrosion [12,13,14,15]. However, in molten halide salts, these oxides either do not form films or form porous layers, unstable due to a very low oxygen activity in molten salts. Consequently, the destruction largely depends on the reactions at interfaces between the molten salt and the surface of the pure metal. In other words, the mechanism of corrosion in molten salts is much more complicated than in aqueous media; the formation of a passivating oxide layer on corrosion-resistant alloys becomes thermodynamically impossible, and therefore the use of many alloys is limited [16,17].

Molten alkali metal fluorides are a promising medium for modern nuclear technologies. The applicability of fluorides is currently limited by the high rates of corrosion and destruction of the metallic materials in them. Traditionally accepted methods of corrosion protection in molten salts, such as alloying metal material, are unacceptable, and it is necessary to develop new methods of corrosion protection in molten salts, such as applying a layer of chemically resistant metal to the surface of candidate materials. The copper coating can significantly reduce corrosion losses.

Contrary to the well-established opinion about the instability of the oxide layer in the melt of halides [18,19,20,21,22,23], in particular fluorides, a number of works have been published [24,25,26,27,28,29] in which there is information about a significant increase in the resistance of the structural material due to the formation of a spinel-type oxide layer on the surface that inhibits the course of the corrosion process.

A coating deposited in a carbonate melt on substrates made of steel and nichromes of various compositions was previously studied [15]. The most optimal composition of the reaction medium for creating an oxide coating of a non-stoichiometric composition, well bonded to the substrate, is the eutectic melt K_2_CO_3_–Na_2_CO_3_–Li_2_CO_3_. Similar coatings were obtained by means of a 4 h exposure of samples at a temperature of 550 °C.

Such coatings could be used as protective coatings in fluoride melts with compositions suitable for MSR only if they are isothermically transferred from one melt to another, which is not technologically feasible. Based on the studied processes of interaction of candidate materials with oxygen-containing compounds in molten salts, we investigated the possibility of the formation of a passivating layer on the surface of candidate materials for MSR directly during corrosion exposure in the FLiNaK melt by setting a certain concentration from 0 to 0.8 wt.% of O^2−^ ions, in the form of Li_2_O. These experiments on the formation of an oxide coating on 12Cr18Ni10Ti steel directly in a fluoride melt showed that, with the range of the concentration of oxygen anions in the melt from 0.2 to 0.4 wt.%, the rate of corrosion of steel decreased by an order of magnitude, which may indicate the detection of the phenomenon of high-temperature passivation of the material, due to modification of the composition of the fluoride melt and reduction in its corrosion activity. It is shown that it is possible to form a protective oxide layer in the melt of alkali metal fluorides, which is the result of the interaction of corrosion products of electronegative steel components and oxygen anions.

Based on previously obtained electrochemical and corrosion data [14], experiments were carried out on stainless steel 12Cr18Ni10Ti with different concentrations of O^2−^ in a fluoride melt to determine the possibility of forming a protective layer during shockless (corrosive) exposure of the material in the melt for 24 h.

## 2. Materials and Methods

### 2.1. Materials

The experiments were performed in a FLiNaK melt with the addition of high-purity lithium oxide with a concentration from 0 to 0.8% by weight by the anion O^2−^. The studies were carried out in an inert argon atmosphere glove box (8.0 ppm in oxygen and 0.1 ppm in moisture). Steel 12Cr18Ni10Ti was used as the test material. The experiments were carried out in parallel on 3 samples at a temperature of 550 °C and an exposure time of 24 h.

### 2.2. Methods

Eutectic melt LiF-NaF-KF (46.5–11.5–42.0 % mol.) was prepared from individual salts of NaF, LiF and KF* HF of the “H.H.” brands; a detailed methodology is presented in [14].

Lithium oxide was synthesized by thermal decomposition of anhydrous lithium hydroxide under vacuum. Lithium hydroxide monohydrate was dehydrated under vacuum at a temperature of 300 °C. Anhydrous LiOH was placed in a magnesium oxide crucible, placed in a sealed quartz tube, and decomposition was carried out at a temperature of 450 °C under vacuum until the release of water ceased. After the water release was finished, the temperature was increased to 800 °C, and the synthesized Li_2_O was treated with hydrogen until the lithium carbonate was completely decomposed. The synthesized lithium oxide was a white powder with a mass fraction of lithium oxide of 99.0% and a Li_2_CO_3_ content of no more than 0.5 wt.%.

Samples of salt melts selected during the experiment, as well as the initial salt composition of FLiNaK and synthesized Li_2_O, were analyzed for impurities using a mass spectrometer with inductively coupled plasma NexIon 2000 (Perkin Elmer, Waltham, MA, USA). The results of the analysis of the initial FLiNaK and Li_2_O are listed in Table 1.

**Figure 1:**
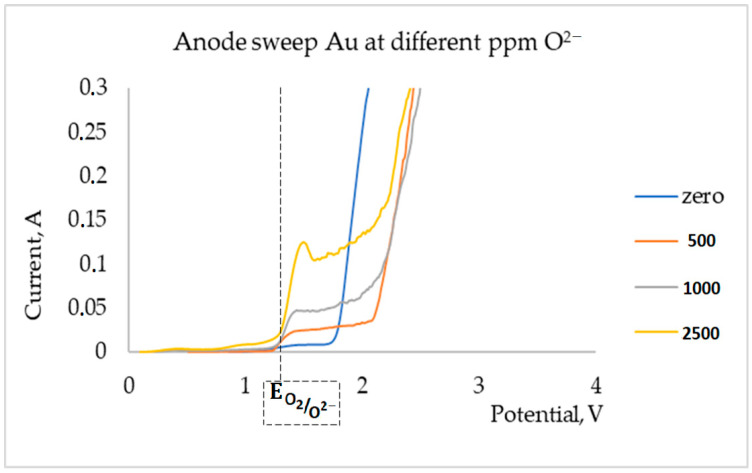
Voltage dependences in a FLiNaK melt with a gold anode.

To determine the number of oxygen anions in the FLiNaK melt, the method of voltampere scanning in the anode region was applied. The AutoLAB PGSTAT 302 N potentiostat (Metrohm Autolab B.V., Utrecht, The Netherlands) was used as a measuring device. The working electrode was a golden ball on a golden wire (with a surface area of 0.88 cm^2^). Molybdenum rods were used as reference for auxiliary electrodes. The scanning speed is 0.5 V/s (to the anode region). The resulting voltage dependences are shown in Figure 1.

According to the obtained voltage dependences, the current increased proportionally to the lithium oxide concentration in the melt. We confirmed the given concentrations of lithium oxide by anodic polarization.

Steel 12Cr18Ni10Ti (Fe-based alloy, 18Cr-10Ni, 0.8 Ti, 0.12 C wt.%) was used as the material for test samples. Before the test, the samples were grounded and polished with abrasive paper of various grain sizes, degreased and dried. After that, their dimensional characteristics were measured with a digital caliper and the mass by the analytical scales AND GR-202.

## 3. Results and Discussion

**Figure 2:**
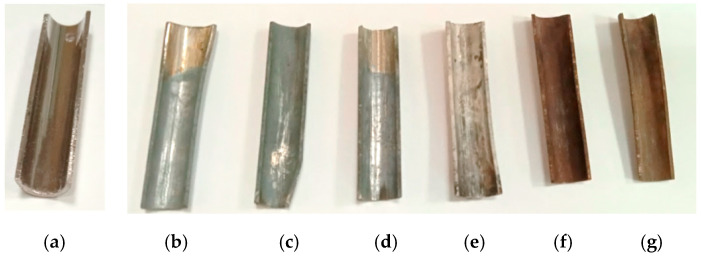
Appearance of the studied samples. (**a**) The original sample; samples were aged in a FLiNaK melt at a concentration of O^2−^: (**b**) 500 ppm, (**c**) 1000 ppm, (**d**) 1500 ppm, (**e**) 2500 ppm, (**f**) 4500 ppm, (**g**) 8000 ppm.

**Figure 3:**
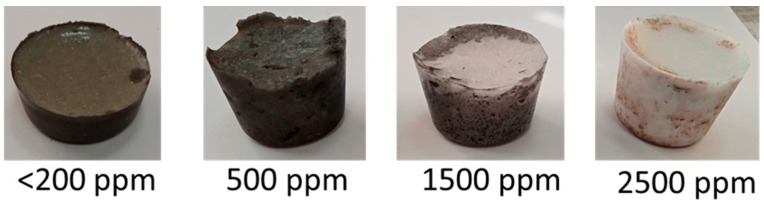
Appearance of the melt after corrosion tests.

Figure 2 shows the appearance of the samples under study, as well as the solidified melt (Figure 3).

It can be noted that, at concentrations up to 2500 ppm, O^2−^ samples have a light gray color; with a further increase in concentration, the samples are covered with a brown coating. In the first case, the color is gray due to selective corrosion; in the second case, the color is brown due to oxide layer formation.

It is also necessary to emphasize the clarification of the melt, i.e., an increase in melt transparency, with an increase in the concentration of lithium oxide in FLiNaK. This trend is associated with a significant decrease in the transition of steel components to the melt.

The corrosion rate obtained from gravimetric and elemental analysis is presented in Table 2.

The corrosion rates obtained from the data of gravimetric and elemental analysis of the salt melt after the end of the experiment have almost identical values for oxygen concentrations in the FLiNaK of up to 1500 ppm O^2−^. For a more accurate gravimetric analysis, washing with pickling solutions, selectively dissolving corrosion products and not damaging the structure of the surface non-corroded layer is required. The selection of such an etching solution requires further research. In this regard, we can say that in this case, the corrosion rates obtained from chemical analysis data are more reliable.

**Figure 4:**
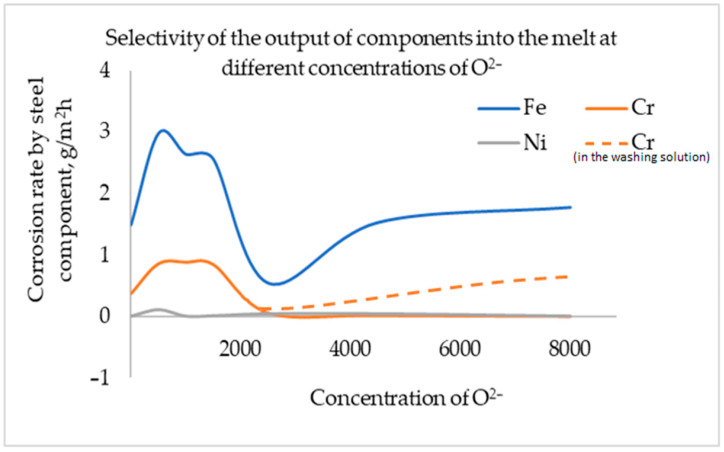
Selectivity of transition of steel components to melt depending on the concentration of lithium oxide additive in FLiNaK, according to elemental analysis.

At a concentration of lithium oxide up to 500 ppm O^2−^, the corrosion rate increases by two times, relative to the similar corrosion exposure in the eutectic FLiNaK mixture without additives. At a concentration of 2500 ppm O^2−^, the corrosion rate is three–four times lower. With a further increase in the oxygen anion content in the melt, the corrosion rate increases again. Figure 4 shows the selectivity of the transition of steel components into the melt depending on the concentration of lithium oxide additives obtained by elemental analysis of the hardened melt after the experiment.

According to chemical analysis, as a result of corrosion exposure, nickel is practically not released in the entire range of lithium oxide concentrations studied in FLiNaK. In turn, at concentrations from 2500 to 4000 ppm O^2−^, the transition of chromium into the melt relative to iron becomes significantly less. In addition, a significant amount of chromium was found in the washing solutions used to remove the salt residue from the samples after an experiment, with a lithium oxide content of over 2500 ppm O^2−^.

The most significant data on changes in the morphology of the surface were obtained using MRSA of cross-section sections. This is due to the fact that it is traditionally assumed that for austenitic stainless steel in halide melts, the prevailing type of corrosion is an intercrystalline one.

**Figure 5:**
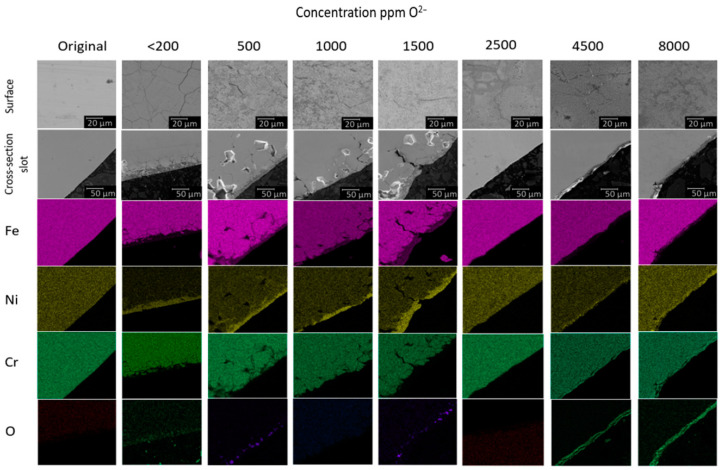
Element mapping of the cross-section of the studied samples of steel 12Cr18Ni10Ti aged in the FLiNaK melt, at different concentrations of Li_2_O.

Figure 5 shows the element mapping of the cross-sectional section of the studied samples of steel 12Cr18Ni10Ti aged in a FLiNaK melt containing various concentrations of Li_2_O.

According to elementary (Figure 4) and MRSA (Figure 5) analyses, it can be noted that, at concentrations above 2500 ppm O^2−^, there is a significant decrease in the yield of chromium into the melt, due to the fact that this component of steel is retained in the oxide surface layer. The concentration of iron in the near-surface layer decreases, while the nickel content does not change. In addition, according to X-ray microanalysis, at concentrations above 2500 ppm O^2−^, there is a change in the nature of corrosion from the typical for halide media, to intercrystalline corrosion, to total corrosion.

At concentrations below 2500 ppm O^2−^, there is a decrease in the concentration of iron and chromium in the near-surface volume of the structural material; it should be assumed that the corrosion products dissolve in the electrolyte without hindrance. In turn, the nickel concentration in it is significantly increased, which indicates the absence and/or slight degradation of the alloy for this component.

Thus, the reactions occurring in the melt system containing lithium oxide/candidate material can be represented as:

Fe^2+^ + O^2−^ = FeO

Ni^2+^ + O^2−^ = NiO

2Cr^3+^ + 3O^2−^ = Cr_2_O_3_

2Fe^3+^ + 3O^2−^ = Fe_2_O_3_

2Ti^3+^ + 3O^2−^ = Ti_2_O_3_

Thus, a decrease in the corrosion rate occurs due to the formation of mixed composition Fe–Cr–O oxides on the surface of the samples, which in turn causes passivation of the shielding type steel due to the solubility of this layer in FLiNaK.

## 4. Conclusions

Based on the obtained electrochemical and corrosion data, the formation of a protective oxygen-containing layer with a thickness of 1 micron was executed at concentrations of 2500 ppm O^2−^.

With the areas of concentration of oxygen anions in the melt from 0.2 to 0.4 wt.%, the rate of corrosion of steel decreases by an order of magnitude, which may indicate the detection of the phenomenon of high-temperature passivation of the material due to modification of the composition of the fluoride melt and reduction in its corrosion activity.

The most dangerous corrosion types, from the point of view of structural reactor materials, are the intercrystalline and pitting types of stainless steel corrosion which occur in fluoride melts. These types of corrosion change to total corrosion when lithium oxide is added due to the “healing” of individual corrosion foci with excess-oxygen-containing compounds (at concentrations of more than 2500 ppm O^2−^).

## Figures and Tables

**Table 1:** Elemental composition of the initial FLiNaK and Li_2_O.

Element	Source Component	
	FLiNaK	Li_2_O
Ti, wt.%	0.0027	0.0007
Cr, wt.%	0.0010	0.0011
Fe, wt.%	0.0032	0.0020
Ni, wt.%	0.0042	0.0014
Mn, wt.%	0.0003	<0.0001
Ca, wt.%	0.0040	0.0050
Co, wt.%	0.0002	<0.0001
Cu, wt.%	0.0018	0.0004
V, wt.%	<0.0001	<0.0001
Zr, mac.%	<0.0001	<0.0001
Mg, mac.%	0.0083	0.0047

**Table 2:** Corrosion rate of 12Cr18Ni10Ti steel samples aged in FLiNaK melt at different concentrations of Li_2_O.

Concentration O^2^^−^		Corrosion Rate, g/m^2^h	
ppm	wt.%	According to Gravimetric Analysis	According to the Data of Elemental Analysis
<200	<0.020	1.448	1.766
500	0.050	3.975	3.936
1000	0.100	3.717	3.516
1500	0.150	3.650	3.404
2500	0.250	0.162	0.541
4500	0.450	0.479	1.589
8000	0.800	0.943	1.705

## References

[1] Komarov V.E., Smolensky V.V., Afonichkin V.K. (2000). Prospects for the use of molten salts in radiochemical technologies. Melts.

[2] LeBlanc D. (2010). Molten salt reactors: A new beginning for an old idea. Nucl. Eng. Des..

[3] Khokhlov V., Ignatiev V., Afonichkin V. (2009). Evaluating physical properties of molten salt reactor fluoride mixtures. J. Fluor. Chem..

[4] Barnes J., Coutts R., Horne T., Thai J. (2019). Characterization of molten salts for application in molten salt reactors. PAM Rev. Energy Sci. Technol..

[5] Magnusson J., Memmott M., Munro T. (2020). Review of thermophysical property methods applied to fueled and un-fueled molten salts. Ann. Nucl. Energy.

[6] Serp J., Allibert M., Benes O., Delpech S., Feynberg O., Ghetta V., Heuer D., Holcomb D., Ignatiev V., Kloosterman J.L. (2014). The molten salt reactor (MSR) in generation IV: Overview and perspectives. Prog. Nucl. Energy.

[7] Williams D.F. (2006). Assessment of Candidate Molten Salt Coolants for the Advanced High-Temperature Reactor (AHTR).

[8] Nuclear Reactors (1956). Part 3. Materials for Nuclear Reactors.

[9] Manly W., Kubs D., de Van D., Douglas D., Inui H., Patriarka P., Roch T., Scott D. (1959). Metallurgical problems associated with the use of molten fluoride systems. Nucl. Fuel React. Mater..

[10] Manly W.D., Adamson G.M., Coobs J.H., DeVan J.H., Douglas D.A., Hoffman E.E., Patriarca P. (1957). Aircraft Reactor Experiment-Metallurgical Aspects.

[11] Ignatiev V.V., Kryukov O.V., Khaperskaya A.V., Kormilitsyn M.V., Kormilitsyna L.A., Semchenkov Y.M., Fedorov Y.S., Feinberg O.S. (2018). Liquid-salt reactor for closing the nuclear fuel cycle for all actinides. At. Energy.

[12] Young D.J. (2016). High Temperature Oxidation and Corrosion of Metals.

[13] Guo S., Zhang J., Wub W., Zhou W. (2018). Corrosion in the molten fluoride and chloride salts and materials development for nuclear applications. Prog. Mater. Sci..

[14] Karfidov E., Nikitina E., Erzhenkov M., Seliverstov K., Chernenky P., Mullabaev A., Tsvetov V., Mushnikov P., Karimov K., Molchanova N. (2022). Corrosion behavior of candidate functional materials for molten salts reactors in LiF–NaF–KF containing actinide fluoride imitators. Materials.

[15] Nikitina E., Karfidov E. (2021). Corrosion of construction materials of separator in molten carbonates of alkali metals. Int. J. Hydrogen Energy.

[16] Wang Y., Zhang S., Ji X., Wang P., Li W. (2018). Material corrosion in molten fluoride salts. Int. J. Electrochem. Sci..

[17] DeVan J.H., Evans R.B. (1962). Corrosion Behavior of Reactor Materials in Fluoride Salt Mixtures.

[18] Janz G.J. (1967). V.A—Melt preparation and purification. Molten Salts Handbook.

[19] Olson L.C., Ambrosek J.W., Sridharan K., Anderson M.H., Allen T.R. (2009). Materials corrosion in molten LiF–NaF–KF salt. J. Fluor. Chem..

[20] Kelleher B.C., Dolan K.P., Brooks P.D., Anderson M., Sridharan K. (2015). Batch-scale hydrofluorination of Li27BeF4 to support molten salt reactor development. J. Nucl. Eng. Radiat. Sci..

[21] Zheng G., Kelleher B., Cao G., Anderson M., Allen T., Sridharan K. (2015). Corrosion of 316 stainless steel in high temperature molten Li2BeF4 (FLiBe) salt. J. Nucl. Mater..

[22] Yang X., Zhang D., Liu M., Feng S., Xue W., Liu H., Yu G., Zhou X., Xia H., Huai P. (2016). Corrosion of SiC induced by Hastelloy N alloy and its corrosion products in LiF–NaF–KF molten salt. Corros. Sci..

[23] De van J.H. (1969). Effect of Alloying Additives of Corrosion Behavior of Nickel-Molybdenum Alloys in Fused Fluoride Mixtures.

[24] Sulejmanovic D., Kurley J.M., Robb K., Raiman S. (2021). Validating modern methods for impurity analysis in fluoride salts. J. Nucl. Mater..

[25] Shen M., Peng H., Ge M., Zuo Y., Xie L. (2015). Use of square wave voltammeter for online monitoring of O2− concentration in molten fluorides at 600 °C. J. Electroanal. Chem..

[26] Ozeryanaya I.N. (1985). Corrosion of metals by molten-salts in heat-treatment processes. Met. Sci. Heat Treat..

[27] Fabre S., Cabet C., Cassayre L., Chamelot P., Delepech S., Finne J., Massot L., Noel D. (2013). Use of electrochemical techniques to study the corrosion of metals in model fluoride melts. J. Nucl. Mater..

[28] Delpech S., Cabet C., Slim C., Picard G.S. (2010). Molten fluorides for nuclear applications. Mater. Today.

[29] Raiman S.S., Lee S. (2018). Aggregation and data analysis of corrosion studies in molten chloride and fluoride salts. J. Nucl. Mater..

